# Probiotic-Enriched Yogurt with Encapsulated *Lacticaseibacillus rhamnosus* and *Bacillus clausii*: Product Characterization and Quality of Life Assessment in Type 2 Diabetes

**DOI:** 10.3390/nu18213523

**Published:** 2026-10-26

**Authors:** Roxana Elena Gheorghita, Delia Viola Reurean Pintilei, Olga Adriana Caliman Sturdza, Roxana Filip, Alina Crina Muresan, Monica Iavorschi, Ioan Ovidiu Sirbu

**Affiliations:** 1 Department of Biochemistry, Victor Babes University of Medicine and Pharmacy Timisoara, 300041 Timisoara, Romania; ovidiu.sirbu@umft.ro; 2 College of Medicine and Biological Science, Stefan cel Mare University of Suceava, 720229 Suceava, Romania; olga.caliman-sturdza@usm.ro (O.A.C.S.); roxana.filip@usm.ro (R.F.); monica.iavorschi@usm.ro (M.I.); 3 Consultmed Medical Center, 700544 Iasi, Romania; 4 Emergency Clinical County Hospital Suceava, 720237 Suceava, Romania; 5 Interdisciplinary Research Centre in the Field of Eco-Nano Technology and Advance Materials CC-ITI, Faculty of Engineering, “Dunarea de Jos” University of Galati, 47 Domneasca Street, 800008 Galati, Romania; alina.muresan@ugal.ro; 6 Center for Complex Network Science, Victor Babes University of Medicine and Pharmacy Timisoara, 300041 Timisoara, Romania

**Keywords:** probiotics, intervention, functional food, health-related quality of life, RAND-36 questionnaire

## Abstract

Background: Probiotic-enriched foods represent a promising nutritional approach for supporting health in chronic metabolic diseases. Yogurt provides a suitable food matrix for probiotic incorporation, while microencapsulation may improve probiotic stability during storage and consumption. Methods: The aim of this study was to develop and characterize a yogurt containing encapsulated *Lacticaseibacillus rhamnosus* (*L. rhamnosus*) and *Bacillus clausii* (*B. clausii*) and to evaluate its effects on selected dimensions of health-related quality of life (HRQoL) in patients with type 2 diabetes mellitus (T2DM). A probiotic-enriched yogurt was prepared using alginate–starch capsules containing *L. rhamnosus* and *B. clausii*. The product was evaluated during three weeks of refrigerated storage for pH, syneresis, water holding capacity, color, and probiotic viability. A randomized, two-arm, placebo-controlled interventional study was conducted in patients with T2DM. Participants received either probiotic yogurt containing encapsulated probiotics or a placebo yogurt containing probiotic-free capsules. HRQoL was assessed at baseline and post-intervention using the RAND 36-Item Health Survey (RAND 36). Results: During three weeks of refrigerated storage, the yogurt showed a progressive decrease in pH and syneresis, while water holding capacity remained indicative of good structural stability. Only minor changes in color were observed, and culturable microorganisms remained recoverable from the capsules during refrigerated storage. In an exploratory, placebo-controlled pilot evaluation, 54 patients with T2DM received two 100 g servings per week for three weeks. After Holm correction for multiple comparisons, the group × time interaction remained significant for vitality (adjusted *p* = 0.004) and emotional well-being (adjusted *p* = 0.009) but not for physical role limitations; in baseline-adjusted ANCOVA (Analysis of Variance), emotional well-being showed the most consistent evidence (adjusted difference 8.3 points, 95% CI 3.5–13.2), whereas results for vitality and physical role limitations were sensitive to model specification. Conclusions: Baseline HRQoL was substantially higher in the probiotic group, limiting causal interpretation. The yogurt showed favorable technological stability with culturable microorganisms persisting in the capsules during storage; the preliminary HRQoL signal requires confirmation in an adequately powered trial.

## 1. Introduction

The human gut is inhabited by a diverse array of bacterial species that perform important metabolic and immune functions, exerting a significant influence on the nutritional and health status of the host. Dietary components also play beneficial roles beyond basic nutrition, leading to the development of the concepts of functional foods and nutraceuticals. The primary role of the diet is to provide sufficient nutrients to meet the basic requirements for maintenance and growth, also contributing to a sense of satisfaction and well-being [1]. Functional foods are classified into three groups according to the way in which they are produced: (i) conventional foods; (ii) modified or fortified foods, and (iii) food ingredients. Conventional foods are whole, unmodified foods (e.g., vegetables, meat, and cereals); fortified foods are conventional foods supplemented with functional food components (e.g., calcium-fortified milk, vitamin-fortified honey); food ingredients are components derived from plants, microorganisms, and other inorganic raw materials that may include macronutrients, essential micronutrients, or non-nutritive components (e.g., inulin, probiotics, iron, etc.) [2].

Probiotics are essential components of food systems, playing an important role in promoting overall health [3]. Their benefits extend beyond digestion, influencing various physiological functions and systemic conditions. This highlights the interplay between probiotics and other biologically active compounds in enhancing health-promoting effects. Probiotics contribute significantly to gut health, immune regulation, and metabolic homeostasis [4]. Their potential is further enhanced when combined with dietary bioactive compounds or incorporated into fermented products, thereby increasing their overall health-promoting effects. Studies have demonstrated that incorporating probiotics into fermented food matrices provides a high intake of biologically active compounds that are important for nutritional interventions and health protection. Accordingly, products such as fruit and vegetables juices, honey and bee products, and, particularly, fermented dairy products have been enhanced through the addition of probiotics [5]. Therefore, the health effects of functional foods are increasingly supported by scientific research. Randomized controlled clinical trials and systematic reviews provide valuable evidence regarding how specific dietary components affect human physiology. These studies have revealed the ways in which such foods influence inflammatory processes, metabolism, hormonal balance, and cardiovascular function [6]. Long-term prospective cohort studies provide particularly strong evidence for the preventive role of functional foods in chronic non-communicable diseases, such as metabolic syndrome, T2DM, certain types of cancer, and neurological disorders [7,8,9,10]. This growing body of evidence provides an important foundation for the development of dietary guidelines and the design of public health strategies.

*Lacticaseibacillus rhamnosus* (*L. rhamnosus*) has been associated with intestinal health, microbial balance, mucosal adhesion, and immune modulation [11,12,13,14]. *Bacillus clausii* (*B. clausii*), a spore-forming probiotic, is characterized by its resistance to acidic conditions and bile, supporting its survival during gastrointestinal transit. It has also been associated with intestinal barrier integrity, immune homeostasis, and pathogen inhibition [15,16]. These complementary characteristics provided the rationale for selecting the two microorganisms for incorporation into a common encapsulation system.

Building on our previous on the development and characterization of sodium-alginate-wheat starch capsules containing *L. rhamnosus* and *B. clausii* [17], the present study extends the research by incorporating the capsules into a yoghurt matrix. Their co-encapsulation was designed to enable their simultaneous delivery to the gastrointestinal tract, potentially combining these complementary actions. Furthermore, our previous results demonstrated that the sodium alginate–starch encapsulation system achieved high encapsulation efficiency (95.83 ± 0.23%) and maintained probiotic viability after encapsulation (more than 96%), while supporting bacterial survival under simulated gastrointestinal conditions (97.05%, cumulative gastric and intestinal exposure) [17]. These findings provided the technological rationale for incorporating the encapsulated strains into yoghurt and investigating this combination in a functional food. Thus, while our previous study focused on capsule properties and probiotic survival under simulated gastrointestinal conditions, the current study evaluates the physicochemical characteristics and probiotic viability of the resulting functional food during refrigerated storage, as well as changes in selected HRQoL domains in patients with T2DM. This approach bridges capsule development and functional food application, providing a preliminary assessment of the product and its potential contribution to patients’ perceived well-being.

Among the most widely used food matrices is yogurt [18], as it provides a suitable environment for maintaining probiotic viability and supporting microbial proliferation. Studies have demonstrated that the addition of probiotics such as *L. rhamnosus*, *Lacticaseibacillus plantarum* (*L. plantarum*), *Lacticaseibacillus acidophilus* (*L. acidophilus*) or *Lacticaseibacillus fermentum* (*L. fermentum*) to dairy products increased cell viability during storage and improved the physicochemical, functional, and sensory properties of the products. Specifically, these products exhibited enhanced viscosity, reduced syneresis, and increased antioxidant activity [19,20,21,22,23,24,25]. Other foods that have been used as probiotic matrices include meat products [26,27], fruit juices [28], fruit-based desserts [29], plant-based beverages [30], chocolate [31], puddings [32], and ice cream [33]. Yogurt is a widely consumed food product worldwide, with the highest consumption reported in Europe. It is produced by fermenting milk with two lactic acid-producing bacteria, *Lactobacillus delbrueckii* subs. *bulgaricus* and *Streptococcus thermophilus*. For most consumers, yogurt is generally considered part of the broader category of fermented dairy products containing various species of *Lacticaseibacillus* or *Bifidum*. Yogurt consumption has been associated with changes in the composition of the human gut microbiome and metabolome [34].

Yogurt is a fermented dairy product mainly made from cow’s milk and is widely appreciated for its nutritional value and beneficial effects on health. Compared with other dairy products, yogurt provides calcium in readily bioavailable form, thereby contributing significantly to meeting daily calcium requirements [35]. Beyond its nutritional contribution, yogurt consumption has been associated with several health benefits, including improved lactose tolerance, support for weight maintenance or weight reduction, a lower percentage of body fat, and beneficial effects attributed to probiotic bacteria. Growing interest in these properties has encouraged the food industry to diversify its product range by developing low-fat and reduced-fat varieties, as well as products containing various additions, such as fruit, chocolate, vanilla, or other flavorings. However, the incorporation of such ingredients often involves the use of food additives, including stabilizers, preservatives, sweeteners, and colorants [36].

## 2. Materials and Methods

### 2.1. Yogurt Preparation and Probiotic Encapsulation

The probiotic strains used in this study were Lacticaseibacillus rhamnosus ATCC 7469 (American Type Culture Collection, Manassas, VA, USA) and Bacillus clausii (SunWave Pharma, Bucharest, Romania). Both strains were cultivated on MRS Agar Vegitone culture medium (Millipore, Sigma Aldrich, Bucharest, Romania). The resulting bacterial suspensions were co-encapsulated in a sodium alginate–starch matrix using the extrusion method previously described by Gheorghita et al. [17]. Briefly, equal volumes of the two probiotic suspensions were combined and incorporated into the biopolymeric solution at a concentration of 20% (*v*/*v*). The capsules were formed by extrusion into a calcium chloride solution. The formulation containing sodium alginate and starch at a 1:1 ratio was selected based on the results of a previous study; the preparation and characterization procedures for the encapsulation system have been described in detail [17]. The alginate–starch capsules (1:1 ratio) containing the co-encapsulated probiotics were added directly to the inoculated milk before fermentation. For each 100 g serving, 10 g of wet capsules were added to 90 g of milk, corresponding to 10% (*w*/*w*) of the final product mass.

The probiotic-enriched yogurt was prepared using whole cow’s milk with an approximate fat content of 3.5% and starter cultures of *Streptococcus thermophilus*, *Lactobacillus acidophilus La-5*, and *Lactobacillus bulgaricus* (Enzymes & Derivates SA, Piatra Neamt, Romania). The milk was homogenized and pasteurized at 65 °C for 30 min. Following pasteurization, the milk was cooled at 40 °C, and the starter culture was added at a concentration of 0.1%. The inoculated milk was subsequently cooled at 30 °C. Alginate–starch capsules (1:1 ratio) containing 20% *L. rhamnosus* and *B. clausii* were prepared according to our previous study [17] and added directly to the inoculated milk at a concentration of 10% before fermentation. The milk containing the starter culture and probiotic capsules was maintained at 30 °C until the pH reached approximately 4.5, at which point fermentation was stopped. The resulting yogurt was packaged in glass jars with screw caps and stored under refrigerated conditions (2–4 °C) for at least 24 h to allow biochemical maturation. According to our previous research, the timing of capsule addition during the yogurt preparation process did not influence the final properties of the product [36]. Subsequently, the product was maintained under refrigerated conditions until further analysis. Participants were instructed to homogenize the yogurt before consumption.

The yogurt used for the refrigerated storage experiment was prepared in a single production batch. In contrast, the yoghurt used in the clinical intervention was prepared before each weekly distribution to the participants. For the storage experiment, the product was initially distributed into 15 glass jars to provide additional samples in case of potential losses and technical issues during analysis. Nine jars were allocated to the planned evaluations. At each storage time point (weeks 1, 2, and 3), three previously unopened jars were used for analyses. Each jar was opened only once and was not reused at subsequent storage time points. Analytical measurements were performed in triplicate, as applicable.

### 2.2. Physicochemical Characterization of Yogurt

The yogurt was evaluated over a three-week period for color, syneresis (S), water-holding capacity (WHC), pH, and other product quality markers.

Syneresis of the yogurt was determined in three weeks of storage. For the evaluation of syneresis, a 100 mL sample was placed in a funnel and subjected to filtration for 6 h. Subsequently, the volume of the liquid collected after filtration was recorded and used for the calculation according to the following formula:

(1) $$S,\;\left(\%\right)=\frac{V1}{V2}\times 100$$

where *V1* represents the volume of whey collected after drainage, and *V2* represents the volume of the yogurt sample.

Water holding capacity (WHC) was evaluated by centrifuging 5 g of yogurt at 4500× *g* for 15 min at 4 °C. The values were calculated using Formula (2), where *W1* represents the mass of whey obtained after centrifugation, and *W2* represents the initial sample mass (5 g).

(2) $$\mathrm{WHC},\;\left(\%\right)=(1-\frac{W1}{W2})\times 100$$

The pH was determined using a Mettler Toledo pH meter (Carl Roth, Karlsruhe, Germany) and the results were expressed as the mean of at least five measurements performed at different locations within the product.

The color of the yogurt during storage was evaluated using a Konika Minolta CR 400 colorimeter calibrated with a standard white plate (L* = 95.46, a* = −0.56, b* = −4). The color parameters L*, a*, b* were recorded using the CIELab color system. Multiple replicates were performed for each sample to ensure the accuracy and reproducibility of the measurements.

### 2.3. Microbiological Analysis and Probiotic Viability

To evaluate the recoverable viable microorganisms in the encapsulated material during refrigerated storage, capsules were manually recovered from the yogurt using sterile forceps and aseptically disrupted to release their contents. The released material was plated on MRS Agar Vegitone plates (Millipore, Sigma Aldrich, Bucharest, Romania) and incubated at 37 °C for 72 h. Following incubation, colonies were counted, and the results were expressed as colony-forming units (CFU) per capsule. The counts represent the combined number of culturable microorganisms recovered from the capsule contents. Scanning electron microscopy (SEM) was performed using a JEOL JSM-T220 A microscope (Oxford Instruments, Abingdon, UK), at an accelerating voltage of 15 kV to examine capsule structure and bacterial morphology. SEM observations were not used to assess bacterial viability.

### 2.4. Clinical Study Design, Participants and HRQoL Questionnaire

The effects of probiotics on well-being were evaluated using a randomized, two-arm, placebo-controlled interventional study, conducted in patients diagnosed with T2DM. Participants were recruited through Consultmed Medical Center Iasi, Romania, under an established collaboration agreement.

**Figure 1:**
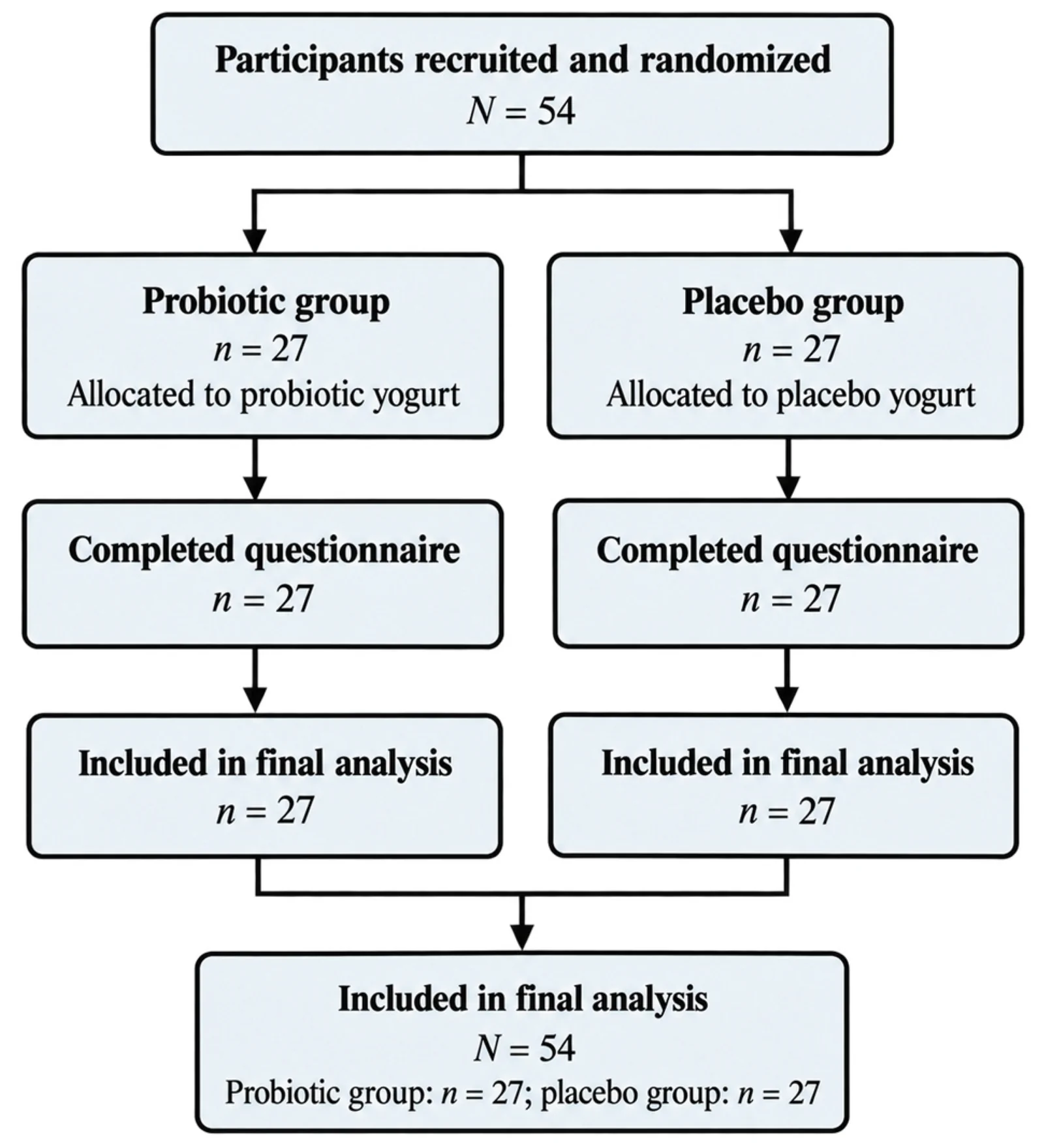
Participant flow diagram.

A total of 54 participants were recruited and randomly allocated to the probiotic and placebo groups, with 27 participants per group. All 54 participants completed the three-week intervention and both assessments and were included in the analysis (Figure 1).

This study design enabled a comparative assessment of the effects of the intervention on well-being. The inclusion criteria for participants were patients with T2DM under the care of a specialist in diabetes, nutrition, and metabolic diseases; age between 25 and 75, and sufficient intellectual capacity to understand and sign the informed consent form. Exclusion criteria were probiotic use within the previous 3 months; antibiotic use within the previous 3 months; comorbid conditions, including primary or secondary immunodeficiencies, cancer, psychiatric disorders, pregnancy, postpartum period, or breastfeeding; eating disorders, including anorexia, bulimia, binge-eating disorder, rumination syndrome, avoidant/restrictive food intake disorder, purging disorder, night eating syndrome, and orthorexia; abuse or dependence on psychotropic substances; alcohol consumption (>150 g alcohol/week); caffeine consumption (>300 mg/day). Eligible participants signed written informed consent prior to study enrollment.

No a priori sample-size calculation was performed. Fifty-four eligible patients were consecutively recruited between February and April 2026, with final follow-up completed in May 2026. The study was not registered in a public trial registry.

Random allocation followed a 1:1 ratio without stratification, using a sequence generated in Microsoft Excel. After completion of the baseline questionnaire, allocation was implemented with sequentially numbered, opaque, sealed envelopes containing codes matching those on the yogurt containers. The code key was held exclusively by the study coordinator, who was not involved in participant allocation or outcome assessment. Treatment assignment was concealed from participants, from the nurse supplying the coded products, and from the physicians who recruited the participants and collected outcome data; both yogurts were identical in appearance and contained capsules, with the added *L. rhamnosus* and *B. clausii* present only in the intervention capsules. The statistician was not blinded.

The intervention consisted of two 100 g servings of the assigned yogurt per week for three weeks, prepared before each weekly distribution. Throughout the study, participants maintained their usual diet, following standard dietary recommendations for T2DM, and their usual physical activity, and were asked to avoid other fermented or potentially prebiotic foods (other yogurts and fermented dairy products, fermented cheese, pickled vegetables). Adherence was assessed by return of the empty jars and by questioning at each weekly visit.

Health-related quality of life was assessed using the RAND 36-Item Health Survey (RAND-36), version 1.0 [37], at baseline, before treatment allocation was revealed, and at the final study visit following the three-week intervention. The instrument was translated from English into Romanian and subsequently back-translated into English before administration and was self-administered without assistance. It covers eight health domains: physical functioning, role limitations due to physical health problems, role limitations due to emotional problems, vitality, emotional well-being, social functioning, bodily pain, and general health perceptions. A separate item assesses perceived change in health compared with one year earlier.

### 2.5. Statistical Analysis

Questionnaire scores were calculated according to the standard methodology, in two stages. In the first stage, the responses were recorded and transformed to a 0–100 scale, with higher scores indicating a more favorable health status. In the second stage, the items corresponding to each domain were averaged to obtain the scores for the eight individual scales. Missing data were excluded from the calculations, and scores were determined as the mean of the available items for each domain. The internal consistency of the questionnaire domains was assessed using Cronbach’s alpha coefficient. For interpretation, values equal or higher than 0.90 were considered excellent, 0.80–0.89 very good, 0.70–0.79 good, 0.60–0.69 acceptable, and values lower than 0.60 poor. The distribution of the continuous variables was assessed using the Shapiro–Wilk test. For between-group comparisons at baseline, independent-samples *t*-tests were used when the data were approximately normally distributed, whereas the Mann–Whitney U test was used otherwise. Fisher’s exact test was used for categorical variables. No individual RAND domain was prespecified as the primary outcome. The domain-level analyses were therefore considered exploratory; the group × time interaction defined the original statistical comparison rather than a prespecified outcome hierarchy. For the eight RAND domains and the separate perceived health-change item, the original analysis used a 2 × 2 mixed-design ANOVA, with group as the between-subject factor and assessment time as the within-subject factor. The group × time interaction assessed between-group differences in change. Effect sizes for the original mixed ANOVA were reported as generalized eta-squared (η^2^G), obtained using the ges option in afex. For the group × time interaction in this two-factor mixed design, η^2^G equals the interaction sum of squares divided by the sum of the interaction sum of squares, the between-subject residual sum of squares, and the within-subject residual sum of squares.

Additional baseline-adjusted analyses were conducted during peer review using ANCOVA, with the follow-up score as the dependent variable and treatment group and the corresponding baseline score as predictors. These analyses were distinct from the original mixed ANOVA, which assessed between-group differences in change. Model diagnostics included visual assessment of baseline–follow-up relationships, homogeneity of regression slopes, residual distributions and variability, and influential observations. HC3 heteroscedasticity-robust standard errors were used for common-slope ANCOVA models. Sensitivity models incorporating a group × baseline interaction were also fitted with HC3 inference to allow different regression slopes between groups. For these interaction models, experimental-minus-control differences were estimated at each outcome’s pooled baseline mean. Nominal 95% confidence intervals and *p*-values used an approximate t reference distribution with 51 residual degrees of freedom for common-slope models and 50 for interaction models.

Floor and ceiling accumulation was quantified as the number and percentage of participants scoring 0 and 100, respectively, in each group at each assessment. Leave-one-participant-out sensitivity analyses were conducted for the interaction models while retaining the full-sample baseline reference values; all participants were retained in the full-sample analyses. Model-specific effects, floor and ceiling frequencies, diagnostics, and leave-one-out summaries are provided in Appendix A, Table A1, Table A2, Table A3, Table A4 and Table A5. These additional analyses were applied consistently across all nine outcomes rather than selecting a model for each outcome based on its *p*-value. The original analyses did not adjust for multiple testing. During revision, Holm adjustment was applied across the nine reported HRQoL outcomes, separately for the original mixed ANOVA interactions and each additional ANCOVA specification. As a sensitivity analysis, Holm adjustment was also applied to the eight RAND domains, treating the perceived health-change item separately (Appendix A, Table A6). These model specifications were used to assess sensitivity rather than as independent confirmatory analyses. Confidence intervals remained nominal 95% intervals; multiplicity adjustment applied to *p*-values only.

Between-group effects from the original analysis were estimated as the mean change from baseline to follow-up in the experimental group minus the corresponding mean change in the control group. Effects were expressed in points on the 0–100 RAND scale, with positive values indicating more favorable change in the experimental group. Nominal 95% confidence intervals were calculated using the t distribution with a pooled variance estimate and 52 degrees of freedom, consistent with the original group × time interaction analysis. These effect estimates were not adjusted for baseline scores.

## 3. Results

### 3.1. Physicochemical Properties of Yogurt During Storage

The resulting product had high biological value, with the following nutritional composition: 4% protein, 3.5% fat, 4.8% carbohydrates, with an energy value of 69 kcal/100 g product (290 kJ/100 g). Based on the recoverable count of approximately 2 × 10^4^ CFU per capsule measured after one week of refrigerated storage and an estimated 100 capsules per 100 g serving, the estimated recoverable microbial count was approximately 2 × 10^6^ CFU per serving. This estimate represents the combined culturable microorganisms recovered from the capsule contents, rather than a separately quantified viable dose of *L. rhamnosus* and *B. clausii*.

Over the three-week testing period, syneresis decreased from 52.00% in week 1 to 42.50% after 21 days of refrigerated storage, while *WHC* increased from 73.81% to 81.56% (Table 1). These changes indicate improved water retention by the yogurt matrix under the specified test conditions.

*WHC* increased progressively over the testing period. These results indicate changes in the water-retention properties of the yogurt matrix during storage. Throughout the testing, both L* and the other color parameters showed only minor changes. Thus, L* decreased from 81.36 during the first week to 76.92 after 21 days of storage (Table 1), indicating a reduction in lightness. The increase in b* values suggests a shift toward more pronounced yellow hues, while the slightly more negative a* values indicate a minor shift toward green hues. These changes may be associated with the release of encapsulated material into the yogurt matrix. The changes in color parameters were less pronounced between the second and third weeks, which may suggest a stabilization trend during storage. Possible explanations include the progression of fermentation and oxidation processes, as well as the gradual release of encapsulated contents. However, these mechanisms were not directly investigated in the present study and should be considered hypotheses. The possible release of probiotics from the encapsulating matrix may also have contributed to the observed color changes.

### 3.2. Microbiological Viability During Refrigerated Storage

**Figure 2:**
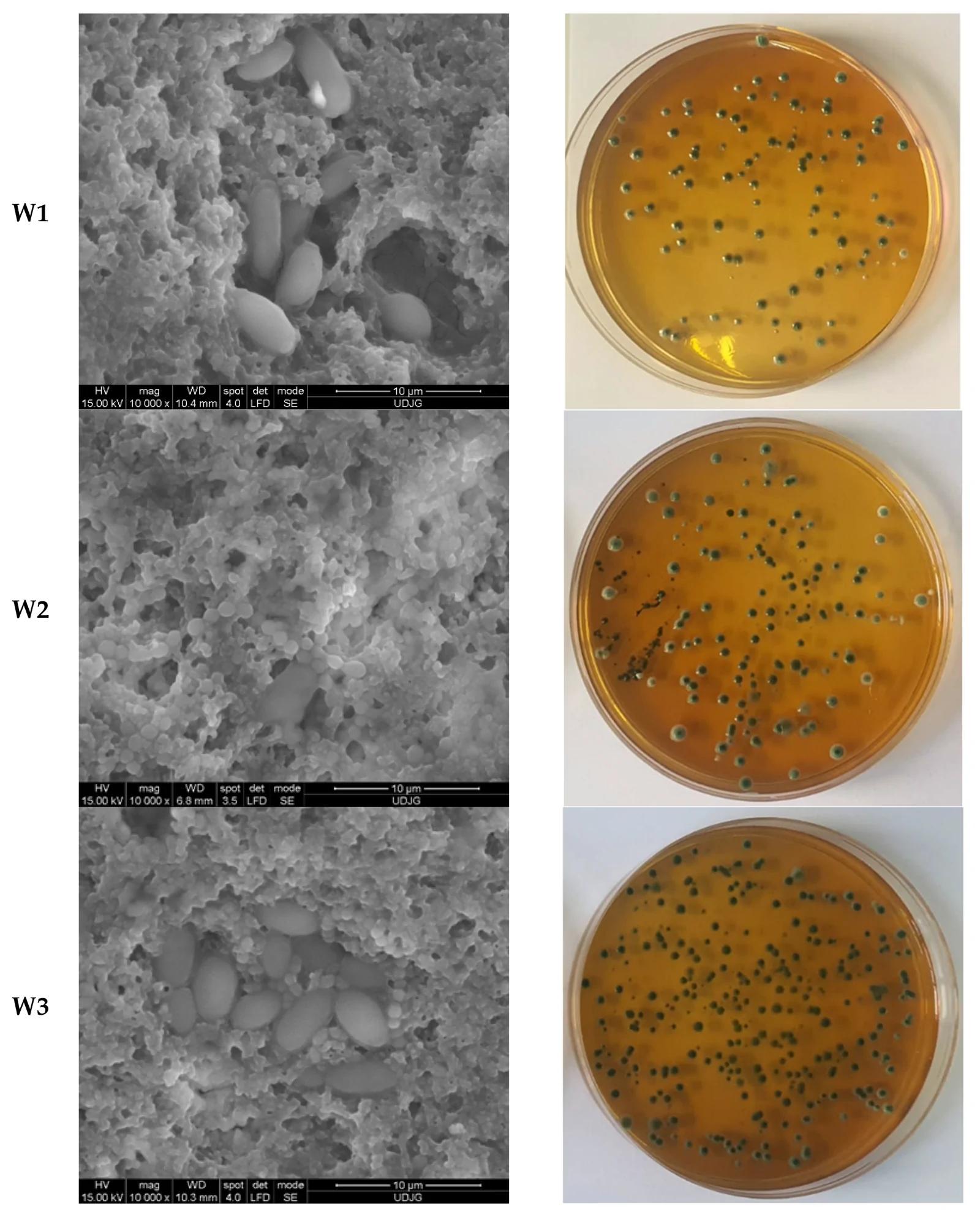
SEM micrographs of capsules recovered from yogurt at weeks 1–3 (W—week of testing).

To evaluate the probiotic viability over a three-week storage period, yogurt samples were analyzed by culture-based enumeration and by evaluating bacterial morphology. The results are presented in Figure 2. The culture-based analysis showed an increase in the number of recoverable culturable microorganisms in the capsules during refrigerated storage, from approximately 20 × 10^3^ CFU/capsule in week 1 to 25 × 10^3^ CFU/capsule in week 2 and 30 × 10^3^ CFU/capsule in week 3. These counts represent the combined culturable microorganisms recovered from the capsule contents.

These findings suggest that the biopolymeric matrix provides a suitable environment for the recovery of culturable microorganisms during the storage period, supporting its potential application in the development of probiotic-enriched functional food.

### 3.3. Changes in RAND-36 Questionnaire Scores After Intervention

At baseline, no statistically significant differences were detected between the probiotic and placebo groups in the measured demographic and anthropometric variables (ag, BMI, sex, and place of residence; Table 2 and Table 3).

The duration of diabetes tended to be longer in the experimental group, although it did not reach the conventional threshold for statistical significance. The duration of diabetes was numerically longer in the experimental group, although the difference did not reach statistical significance (Table 2).

Following the baseline comparisons (Table 3), the analysis proceeded to examine the study parameters and QoL scores derived from the RAND-36 questionnaire.

All participants were receiving metformin; 32 were also treated with an SGLT2 inhibitor, 24 with a GLP-1 receptor agonist and 2 with basal insulin. All received an ACE inhibitor or angiotensin-receptor blocker and a statin; 17 received low-dose aspirin and 32 a fibrate (Table 4). Eligibility required that therapy had been stable for at least three months before enrolment, and no change in medication was reported during the intervention.

Background therapy was not a pre-specified study variable and was retrieved from the clinic records for the whole cohort; a breakdown by allocation group is not available. Categories are not mutually exclusive. Therapy had been stable for at least three months before enrolment and remained unchanged during the intervention.

All participants consumed all six servings. Adverse events were actively solicited at each weekly visit and at the final visit; none were reported in either group. No participant withdrew from the study.

Table 5 presents the Cronbach’s α analysis used to assess the internal consistency of the questionnaire domains.

The results obtained for the analyzed domains of the RAND-36 questionnaire showed good to very good internal consistency. Cronbach’s α values ranged from 0.739 to the vitality domain to 0.898 for physical functioning, supporting the use of these composite scores in subsequent analysis. To identify the most appropriate statistical test, the two groups were compared based on the information obtained from the first questionnaire administered (Table 6).

At baseline, the experimental group had higher scores across all eight RAND domains and the separate perceived health-change item (Table 6). These differences motivated additional analyses of follow-up scores adjusted for the corresponding baseline score. The original group × time interaction compares changes between groups but is not equivalent to baseline-score adjustment.

Analysis of the scores obtained by the two groups for each questionnaire domain, assessed at both baseline and post-intervention, showed that the experimental group had higher scores at baseline (Table 7).

The original 2 × 2 mixed ANOVA assessed between-group differences in change through the group × time interaction (Table 8), and additional baseline-adjusted ANCOVA results are reported separately in Table 9.

In the original mixed ANOVA, the unadjusted group × time interactions were significant for physical role limitations (F (1,52) = 5.403, *p* = 0.024), vitality (F (1,52) = 13.856, *p* < 0.001), and emotional well-being (F (1,52) = 11.785, *p* = 0.001). After Holm adjustment across the nine HRQoL outcomes, the interactions remained significant for vitality (adjusted *p* = 0.0044) and emotional well-being (adjusted *p* = 0.0094), but not for physical role limitations (adjusted *p* = 0.1683).

The estimated experimental-minus-control differences in change were 14.81 points for physical role limitations (nominal 95% CI 2.02–27.60), 9.63 points for vitality (4.44–14.82), and 5.93 points for emotional well-being (2.46–9.39). The remaining nominal confidence intervals included zero. These estimates describe unadjusted differences in change; the wide interval for physical role limitations indicates considerable uncertainty about its magnitude.

All 54 participants were included in the additional baseline-adjusted analyses. Diagnostic assessment of the common-slope models indicated greater residual variability in the experimental group, departures from residual normality, and potentially influential observations (Appendix A, Table A4). In the sensitivity models with HC3 inference, evidence of a group × baseline interaction was observed for physical role limitations (*p* = 0.0008) and the separate perceived health-change item (*p* = 0.0164). These diagnostic interaction *p*-values were unadjusted and do not test the intervention effect at the baseline reference score (Appendix A, Table A3).

In common-slope ANCOVA with HC3 inference (Table 9), adjusted experimental-minus-control differences were 24.31 points for physical role limitations (nominal 95% CI 7.27–41.34), 11.59 points for vitality (2.97–20.20), and 8.33 points for emotional well-being (3.49–13.18). After Holm adjustment across nine outcomes, *p*-values were 0.0484, 0.0659, and 0.0101, respectively. In models allowing different slopes, the corresponding effects at pooled baseline means were 23.28, 10.39, and 7.58 points, with Holm-adjusted *p*-values of 0.0119, 0.0503, and 0.0089 (Appendix A, Table A1). For vitality, 0.0503 is approximately 0.05 but slightly above the threshold; the evidence was therefore sensitive to model specification.

Marked floor and ceiling accumulation was observed. For physical role limitations, 14/27 control participants (51.9%) scored 0 at baseline and 15/27 (55.6%) at follow-up; in the experimental group, 12/27 (44.4%) scored 100 at baseline and 14/27 (51.9%) at follow-up. For emotional role limitations, 19/27 experimental participants (70.4%) scored 100 at baseline and 21/27 (77.8%) at follow-up. For social functioning, the corresponding experimental-group ceiling frequencies were 11/27 (40.7%) and 15/27 (55.6%) (Appendix A, Table A2).

In leave-one-participant-out analyses of the different-slopes models, physical role limitations, vitality, and emotional well-being retained positive estimates and nominal confidence intervals excluding zero in all 54 iterations. Effect estimates ranged from 19.72 to 25.03, 7.64 to 11.33, and 5.80 to 8.05 points, respectively (Appendix A, Table A5). These sensitivity results were not multiplicity-adjusted.

When Holm adjustment was restricted to the eight RAND domains, vitality had adjusted *p*-values of 0.0431 in the different-slopes model and 0.0564 in the common-slope model, illustrating sensitivity to both model specification and family definition (Appendix A, Table A5).

**Figure 3:**
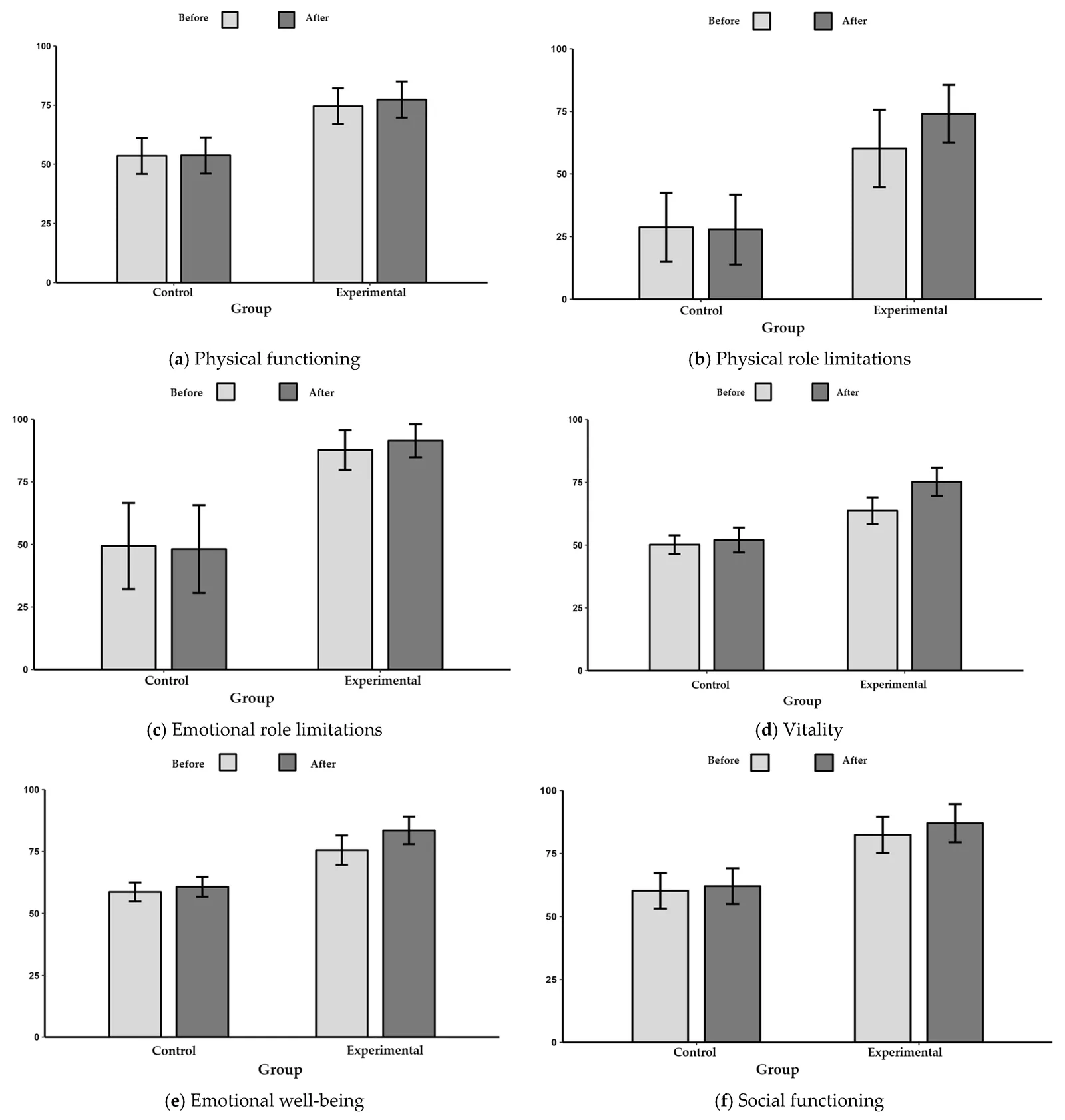

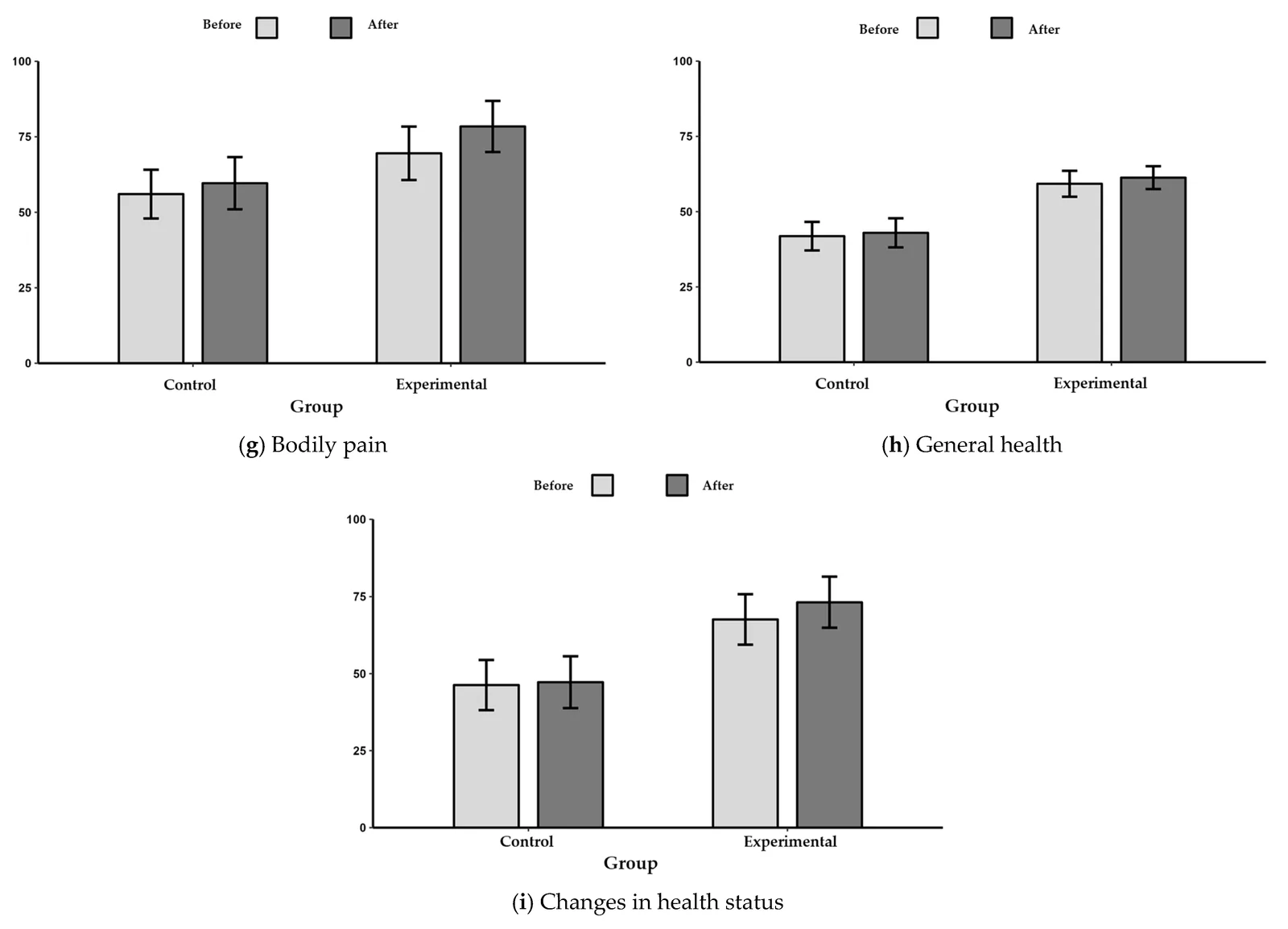
Scores obtained for each domain assessed using the questionnaire. (Bars show the mean score in each group at each assessment, with error bars representing 95% confidence intervals for those means; between-group effect estimates and their nominal confidence intervals are reported separately in Table 8 and Table 9).

The scores obtained for each domain, compared between the groups at the two intervention time points, are presented in Figure 3.

## 4. Discussion

The results obtained from testing the developed product over the three-week period showed the potential of sodium alginate and starch for probiotic encapsulation, as well as the suitability of natural yogurt as a matrix for their incorporation. During the testing period, only minor changes in color were observed, with the product showing greater stability after the second week (Table 1). The pH decreased through the testing period, from 4.51 to 4.32. Similar effects were reported by Kailasapathy. In his study, the encapsulation of *L. acidophilus* and *B. lactis* in sodium alginate and starch, followed by their incorporation into natural yogurt, promoted an increase in acidity due to the acid-producing activity pf the probiotics. However, in his study, the pH decreased below 4.2, which resulted in whey separation and increased syneresis [38], an effect that was not observed in the present study. The decrease in pH observed during refrigerated storage may be associated with ongoing acidification process in the yogurt matrix. Changes in pH can also be attributed to the fermentation of sugars, which during the lactic acid bacteria used for milk fermentation and yogurt production produce lactic acid at concentrations ranging between 0.6% and 1.0%, as well as volatile compounds such as acetaldehyde and diacetyl. These compounds contribute to the characteristic sensory properties of yogurt and also contribute to product shelf-life extension [39]. Furthermore, studies evaluating the acidity of probiotics added to yogurt in encapsulated and non-encapsulated forms have reported smaller variations in pH in yogurt containing free probiotic strains over a seven-week testing period. Color also showed only minor variations when samples containing strains encapsulated in alginate and starch were evaluated [38].

The high WHC values observed during storage indicate favorable water-retention properties under the applied centrifugated conditions. Similar results were obtained in another study investigating the development of functional yogurt through the addition of *B. lactis B99* encapsulated in sodium alginate [40].

The encapsulation of the probiotic strains and the enrichment of the yogurt showed favorable effects in the present study. The results indicate that culturable microorganisms remained recoverable from the capsules during refrigerated storage (Figure 2), which is of particular importance for the development of a functional food product.

Several studies have reported reductions in probiotic counts following encapsulation and incorporation into food products. For example, Ajlouni et al. observed a reduction in the encapsulated probiotic count after 21 days following their addition to yogurt. In contrast, the total probiotic count increased during in vitro colonic fermentation, indicating high bioavailability at the colonic level [40]. The incorporation of *L. casei* into biopolymers favored cell survival through a 21-day testing period in yogurt stored under refrigerated conditions [39].

The HRQoL analyses suggested favorable changes in selected domains, with the most consistent multiplicity-adjusted evidence observed for emotional well-being. Findings for vitality and physical role limitations depended on whether change scores or baseline-adjusted follow-up scores were analyzed. In the original mixed ANOVA, Holm-adjusted group × time interactions remained significant for vitality and emotional well-being, whereas the physical role-limitations interaction did not remain significant after adjustment (adjusted *p* = 0.1683). These findings are consistent with previous evidence showing that probiotic-containing yogurt may positively influence HRQoL. In a randomized controlled trial involving 961 women, Kinoshita et al. reported significant improvements in general health and vitality after 16 weeks of daily consumption of probiotic yogurt [41]. For physical role limitations, the mean score in the experimental group increased from 60.19 to 74.07, whereas the control group showed a slight decrease from 28.70 to 27.78. Similarly, vitality increased from 63.70 to 75.19 in the experimental group, compared with a smaller increase from 50.19 to 52.04 in the control group. Emotional well-being also showed a more pronounced improvement in the experimental group, with mean scores increasing from 75.56 to 83.56, compared with an increase from 58.67 to 60.74 in the control group. The between-group differences in change provide a measure of the magnitude of the observed changes on the 0–100 RAND scale, rather than relying exclusively on statistical significance. The estimated difference for physical role limitations was 14.81 points, but its nominal confidence interval ranged from 2.02 to 27.60 points, indicating substantial uncertainty. The corresponding estimates were 9.63 points for vitality and 5.93 points for emotional well-being. Statistical significance alone does not establish clinical importance; these estimates should be interpreted alongside baseline imbalance, multiplicity, and the limitations of the measurement scales.

The numerical changes were largest in physical role limitations, vitality, and emotional well-being, but the strength of statistical evidence differed across domains and analytical models. The results are consistent with previous evidence suggesting that dietary interventions may influence health-related quality of life, particularly dimensions related to vitality and physical role limitations. Thus, in the Women’s Health Initiative Modification Trial, Assaf et al. reported improvements in physical role limitations, vitality, and general health after one year of dietary intervention [42]. The present study showed favorable numerical changes in these domains, although conclusions about the intervention require consideration of multiplicity and baseline-adjusted analyses. For physical, emotional and social functioning, bodily pain, and perceived change in health status, the observed numerical changes were not accompanied by statistically significant group × time interactions. Nevertheless, the experimental group generally showed a positive direction of change across these domains. The results do not support a generalized HRQoL improvement or an unqualified claim of significant improvement in three domains; emotional well-being showed the most consistent evidence, while vitality and physical role limitations were sensitive to the analytical approach. The findings are consistent with previous evidence suggesting that probiotic-based interventions may influence selected dimensions of HRQoL in patients with T2DM. Horvath et al. reported a significant improvement in the physical role limitations domain when using the RAND-36 questionnaire after three months of multispecies symbiotic supplementation, with a mean change of 5.4 points in the experimental group, compared with −5 points in the placebo group [43]. Although the intervention and duration differed, both studies indicate that probiotic or symbiotic supplementation may be associated with improvements in selected physical and functional dimensions of HRQoL. Of particular relevance to the present study, Darwiche et al. evaluated HRQoL in 30 patients with T2DM using the RAND-36 questionnaire. After 12 weeks of Nordic dietary intervention, significant improvements were reported in physical role limitations, general health, vitality, social functioning, and mental health, with the largest increases observed for physical and social functioning [44]. The direction of the numerical changes overlaps with some domains reported in previous studies, but differences in intervention, duration, baseline distributions, and statistical analyses limit direct comparisons. Despite differences in dietary approach and study design, the use of the same instrument and the inclusion of patients with T2DM provide a useful context for interpreting these results. Comparisons with previous probiotic and dietary intervention studies should be interpreted cautiously. The present intervention involved two 100 g servings per week for three weeks, whereas the cited studies differed in duration, consumption schedule, microbial formulation, population, or the extent of dietary modification. These differences limit direct comparison of the findings and do not establish the efficacy of the present formulation. Interpretation is further constrained by the substantial baseline imbalance in HRQoL scores and uncertainty regarding the species-specific viable dose administered. The clinical findings should therefore be considered exploratory. Interpretation is limited by substantial baseline imbalance, unequal residual variability, and floor and ceiling accumulation, particularly in the role-limitation domains. Some fitted values from the common-slope models fell outside the possible 0–100 score range, underscoring limitations of the linear approximation. HC3 inference addresses heteroscedasticity but does not remove these measurement constraints or potential model misspecification. The effect for physical role limitations varied with baseline score, and estimates from the different-slopes model therefore apply at the stated reference score. For general health, the observed baseline ranges overlapped only between 40 and 55 points. Although leave-one-out analyses supported the stability of the three highlighted point estimates, they do not replace multiplicity adjustment or establish clinical importance. Overall, emotional well-being showed the most consistent evidence after multiplicity adjustment across the original and baseline-adjusted analyses. Physical role limitations were supported by the baseline-adjusted models but not by the original mixed ANOVA after Holm adjustment. Vitality remained significant in the original analysis, while the different-slopes ANCOVA yielded an adjusted *p*-value of 0.0503, approximately 0.05 but slightly above the conventional threshold; the common-slope ANCOVA yielded 0.0659. Accordingly, evidence for vitality was sensitive to model specification. These findings warrant cautious interpretation rather than an unqualified claim of significant improvement in three domains.

The study was not prospectively registered, and no separate, prospectively documented statistical analysis plan was prepared. This limits independent verification of the prespecification of outcomes and analyses. No a priori sample-size calculation was performed, and the sample was determined by recruitment during the study period. Several RAND-36 items refer to the preceding four weeks. Consequently, assessment after a three-week intervention includes part of the pre-intervention period, limiting the temporal specificity of the follow-up scores. The separate health-transition item compares current health with health one year earlier and should not be interpreted as a specific measure of change attributable to the three-week intervention. Background medication is reported for the whole cohort only, as it was not a pre-specified study variable; although the regimen was homogeneous (all participants on metformin, a renin–angiotensin inhibitor and a statin, with stable therapy before and during the study), an imbalance in second-line glucose-lowering agents between groups cannot be formally excluded.

## 5. Conclusions

The developed probiotic yogurt showed favorable physicochemical stability and maintained recoverable culturable microorganisms in the encapsulated material during refrigerated storage. In the clinical evaluation, emotional well-being showed the most consistent evidence across multiplicity-adjusted analyses. Findings for physical role limitations and vitality were sensitive to baseline adjustment and model specification, and do not support an unqualified claim of significant improvement in three HRQoL domains. The relatively small sample size, short intervention period, baseline differences, and floor and ceiling accumulation limit interpretation. In addition, the storage experiment was conducted using a single production batch, which limits conclusions regarding the reproducibility of the product’s physicochemical and microbiological characteristics across independent production batches. Larger studies with prespecified outcomes, independent production batches, and longer follow-up are needed to assess the clinical relevance and durability of these findings.

## 6. Patents

The novel product developed in this research is the subject of a patent application entitled *Yogurt with Encapsulated Lacticaseibacillus rhamnosus and Bacillus clausii and Process for Obtaining the Same*, filed with the Romanian State Office for Inventions and Trademarks (OSIM) under application No. A00200/02.04.2026 and with the European Patent Office (EPO) under application 26464006.1/EP26464006/11.08.2026.

## Figures and Tables

**Table 1:** Evaluation of yogurt parameters during storage.

Week	pH	*S* (%)	*WHC* (%)	Color		
				L*	a*	b*
1	4.51 ± 0.02	52.00 ± 0.01	73.81 ± 0.01	81.36 ± 0.03	−2.49 ± 0.02	7.91 ± 0.01
2	4.46 ± 0.01	48.00 ± 0.01	76.74 ± 0.01	78.14 ± 0.02	−2.53 ± 0.03	8.14 ± 0.07
3	4.32 ± 0.01	42.50 ± 0.01	81.56 ± 0.01	76.92 ± 0.04	−2.59 ± 0.08	8.46 ± 0.01

Values are expressed as mean ± standard deviation, calculated from the measurements obtained of three independent jars at each storage time point; *S*—syneresis, *WHC*—water holding capacity.

**Table 2:** Baseline comparison of continuous variables between two study groups.

Variabile	Control	Experimental	Test	*p*
Age (yrs)	54.78 ± 16.24	60.70 ± 9.14	Test T Independent	0.106
BMI (kg/m^2^)	29.32 [25.89–31.94]	30.04 [27.47–33.73]	Mann–Whitney U	0.228
Duration of diabetes (yrs)	5.00 [3.00–9.00]	8.00 [4.50–17.50]	Mann–Whitney U	0.055

Continuous variables were reported ad mean ± standard deviation when the distribution was approximatively normal, and as median [Q1–Q3] when the distribution was non-normal. Between-group comparisons were performed using the *t*-test or Mann–Whitney U test, depending on the results of the Shapiro–Wilk normality test.

**Table 3:** Categorical variables at baseline.

Variable		Control	Experimental	Test	*p*
Sex	Female	19 (70.4%)	15 (55.6%)	Fisher’s exact	0.398
	Male	8 (29.6%)	12 (44.4%)		
Place of residence	Rural	11 (40.7%)	8 (29.6%)	Fisher’s exact	0.569
	Urban	16 (59.3%)	19 (70.4%)		

**Table 4:** Background pharmacological therapy at enrolment (whole cohort, *n* = 54).

Therapy	*n* (%)
Glucose-lowering therapy	
Metformin	54 (100)
SGLT2 inhibitor	32 (59.3)
GLP-1 receptor agonist	24 (44.4)
Basal insulin	2 (3.7)
Cardiovascular therapy	
ACE inhibitor or angiotensin-receptor blocker	54 (100)
Statin	54 (100)
Fibrate	32 (59.3)
Low-dose aspirin	17 (31.5)

ACE—angiotensin-converting enzyme; GLP-1—glucagon-like peptide-1; SGLT2—sodium–glucose cotransporter 2.

**Table 5:** Internal consistency of the questionnaire domains.

Category	Items	Cronbach α	Interpretation
Physical functioning	10	0.898	Very good
Physical role limitations	4	0.842	Very good
Emotional role limitations	3	0.854	Very good
Vitality	4	0.739	Good
Emotional well-being	5	0.850	Very good
Social functioning	2	0.740	Good
Bodily pain	2	0.881	Very good
General health	5	0.744	Good

**Table 6:** Baseline comparisons between the study groups.

Category	Control Group	Experimental Group	*p*	Test
Physical functioning	50.00 [40.00–75.00]	75.00 [62.50–90.00]	0.001	Wilcoxon
Physical role limitations	0.00 [0.00–50.00]	50.00 [25.00–100.00]	0.006	Wilcoxon
Emotional role limitations	33.33 [0.00–100.00]	100.00 [66.67–100.00]	0.002	Wilcoxon
Vitality	50.19 ± 9.85	63.70 ± 14.05	<0.001	Test T
Emotional well-being	56.00 [52.00–66.00]	80.00 [64.00–88.00]	<0.001	Wilcoxon
Social functioning	62.50 [50.00–75.00]	87.50 [75.00–100.00]	<0.001	Wilcoxon
Bodily pain	55.00 [45.00–72.50]	67.50 [48.75–88.75]	0.041	Wilcoxon
General health	50.00 [37.50–50.00]	55.00 [55.00–65.00]	<0.001	Wilcoxon
Changes in health status	50.00 [25.00–50.00]	75.00 [50.00–75.00]	0.001	Wilcoxon

**Table 7:** Analysis of the scores obtained y both groups at baseline and post-intervention.

Category	Control Group		Experimental Group	
	Initial	Final	Initial	Final
Physical functioning	53.52 ± 20.28	53.70 ± 20.36	74.63 ± 20.04	77.41 ± 20.26
Physical role limitations	28.70 ± 36.50	27.78 ± 36.91	60.19 ± 41.17	74.07 ± 30.60
Emotional role limitations	49.38 ± 45.64	48.15 ± 46.53	87.65 ± 20.98	91.36 ± 17.52
Vitality	50.19 ± 9.85	52.04 ± 13.10	63.70 ± 14.05	75.19 ± 14.77
Emotional well-being	58.67 ± 10.17	60.74 ± 10.64	75.56 ± 15.74	83.56 ± 14.77
Social functioning	60.19 ± 18.68	62.04 ± 18.82	82.41 ± 19.07	87.04 ± 20.06
Bodily pain	56.02 ± 21.41	59.63 ± 22.91	69.54 ± 23.45	78.43 ± 22.43
General health	41.85 ± 12.57	42.96 ± 12.80	59.26 ± 11.41	61.30 ± 10.06
Changes in health status	46.30 ± 21.60	47.22 ± 22.29	67.59 ± 21.72	73.15 ± 21.85

**Table 8:** Original mixed ANOVA group × time interactions and between-group differences in change.

Outcome	F (1, 52)	η^2^G	Difference in Change [95% CI]	Unadjusted *p*	Holm-Adjusted *p*
Physical functioning	1.605	0.0011	2.59 [−1.51, 6.70]	0.2109	1.0000
Physical role limitations	5.403	0.0106	14.81 [2.02, 27.60]	0.0240	0.1683
Emotional role limitations	1.600	0.0013	4.94 [−2.90, 12.77]	0.2115	1.0000
Vitality	13.856	0.0340	9.63 [4.44, 14.82]	<0.001	0.0044
Emotional well-being	11.785	0.0132	5.93 [2.46, 9.39]	0.0012	0.0094
Social functioning	1.029	0.0014	2.78 [−2.72, 8.27]	0.3152	1.0000
Bodily pain	2.292	0.0035	5.28 [−1.72, 12.27]	0.1361	0.8168
General health	0.338	0.0004	0.93 [−2.27, 4.12]	0.5634	1.0000
Perceived health change (single item)	0.871	0.0029	4.63 [−5.32, 14.58]	0.3549	1.0000

Effects are expressed in points on the 0–100 RAND scale and equal the mean change (follow-up—baseline) in the experimental group minus that in the control group; positive values indicate more favorable change in the experimental group. η^2^G denotes generalized eta-squared. All interaction tests have 1 and 52 degrees of freedom. Confidence intervals are nominal 95% intervals based on pooled-variance t inference. Estimates are not baseline-adjusted. Holm-adjusted *p*-values refer to the nine reported HRQoL outcomes; perceived health change is a single item, not a ninth domain.

**Table 9:** Additional baseline-adjusted common-slope ANCOVA with HC3 inference.

Outcome	Adjusted Difference [95% CI]	Unadjusted *p*	Holm-Adjusted *p*
Physical functioning	3.91 [−0.86, 8.67]	0.1059	0.4236
Physical role limitations	24.31 [7.27, 41.34]	0.0060	0.0484
Emotional role limitations	8.44 [−5.05, 21.94]	0.2149	0.4298
Vitality	11.59 [2.97, 20.20]	0.0094	0.0659
Emotional well-being	8.33 [3.49, 13.18]	0.0011	0.0101
Social functioning	5.25 [−5.54, 16.04]	0.3335	0.4298
Bodily pain	7.35 [−1.87, 16.56]	0.1156	0.4236
General health	3.69 [0.63, 6.75]	0.0190	0.1143
Perceived health change (single item)	11.76 [0.74, 22.78]	0.0369	0.1845

The model was follow-up score ~ group + corresponding baseline score. Effects are experimental minus control, in RAND points, under a common-slope assumption. Confidence intervals are nominal 95% intervals based on HC3 standard errors and approximate t inference with 51 residual degrees of freedom. Holm adjustment was applied across the nine HRQoL outcomes. Models allowing different slopes are reported in Appendix A, Table A1; their effects are conditional on the stated baseline reference score.

## Data Availability

The original contributions presented in this study are included in the article. Further inquiries can be directed to the corresponding authors.

## Abbreviations

The following abbreviations are used in this manuscript:

ACE	Angiotensin-Converting Enzyme
ANOVA	Analysis of Variance
BMI	Body Mass Index
CFU	Colony Forming Units
GLP-1	Glucagon-like Peptide-1
HRQoL	Health-Related Quality of Life
L	Luminosity
QoL	Quality of Life
RAND 36	RAND 36-Item Health Survey
S	Syneresis
SD	Standard Deviation
SGLT2	Sodium–glucose Cotransporter 2
T2DM	Type 2 Diabetes Mellitus
WHC	Water-Holding Capacity

## Appendix A

Sensitivity analyses and diagnostic summaries for the HRQoL analyses.

All full-sample analyses included 54 participants (27 per group). The eight RAND domains and the single perceived health-change item were scored on a 0–100 scale, with higher values indicating more favorable health. Common-slope ANCOVA is reported in Table 8. This appendix documents the additional sensitivity analyses and diagnostics. All effect confidence intervals are nominal; multiplicity adjustment applies to *p*-values only.

**Table A1:** Baseline-adjusted ANCOVA allowing different slopes, with HC3 inference.

Outcome	Baseline Reference	Difference [95% CI]	Unadjusted *p*	Holm-Adjusted *p*
Physical functioning	64.07	3.92 [−1.43, 9.28]	0.1475	0.4197
Physical role limitations	44.44	23.28 [9.38, 37.18]	0.0015	0.0119
Emotional role limitations	68.52	15.69 [−3.39, 34.78]	0.1049	0.4197
Vitality	56.94	10.39 [2.94, 17.83]	0.0072	0.0503
Emotional well-being	67.11	7.58 [3.23, 11.93]	<0.001	0.0089
Social functioning	71.30	5.22 [−6.74, 17.17]	0.3850	0.4197
Bodily pain	62.78	7.15 [−2.41, 16.71]	0.1393	0.4197
General health	50.56	3.98 [0.68, 7.28]	0.0190	0.1140
Perceived health change (single item)	56.94	11.72 [1.60, 21.84]	0.0240	0.1202

The model was follow-up score ~ group + centered baseline + group × centered baseline. Baseline was centered at the pooled full-sample mean for each outcome. Differences are experimental minus control at the stated baseline reference score, not assumed constant across baseline values. HC3 standard errors and approximate t inference with 50 residual degrees of freedom were used. Holm adjustment refers to the nine HRQoL outcomes. For vitality, adjusted p = 0.0503 is approximately 0.05, but the unrounded value exceeds 0.05.

**Table A2:** Floor and ceiling frequencies by group and assessment.

Outcome	Group	Baseline 0	Baseline 100	Follow-Up 0	Follow-Up 100
Physical functioning	Control	0 (0.0%)	0 (0.0%)	0 (0.0%)	0 (0.0%)
Physical functioning	Experimental	0 (0.0%)	6 (22.2%)	0 (0.0%)	7 (25.9%)
Physical role limitations	Control	14 (51.9%)	4 (14.8%)	15 (55.6%)	4 (14.8%)
Physical role limitations	Experimental	6 (22.2%)	12 (44.4%)	0 (0.0%)	14 (51.9%)
Emotional role limitations	Control	10 (37.0%)	11 (40.7%)	11 (40.7%)	11 (40.7%)
Emotional role limitations	Experimental	0 (0.0%)	19 (70.4%)	0 (0.0%)	21 (77.8%)
Vitality	Control	0 (0.0%)	0 (0.0%)	0 (0.0%)	0 (0.0%)
Vitality	Experimental	0 (0.0%)	1 (3.7%)	0 (0.0%)	2 (7.4%)
Emotional well-being	Control	0 (0.0%)	0 (0.0%)	0 (0.0%)	0 (0.0%)
Emotional well-being	Experimental	0 (0.0%)	1 (3.7%)	0 (0.0%)	6 (22.2%)
Social functioning	Control	0 (0.0%)	0 (0.0%)	0 (0.0%)	0 (0.0%)
Social functioning	Experimental	0 (0.0%)	11 (40.7%)	0 (0.0%)	15 (55.6%)
Bodily pain	Control	0 (0.0%)	1 (3.7%)	0 (0.0%)	2 (7.4%)
Bodily pain	Experimental	0 (0.0%)	6 (22.2%)	1 (3.7%)	7 (25.9%)
General health	Control	0 (0.0%)	0 (0.0%)	0 (0.0%)	0 (0.0%)
General health	Experimental	0 (0.0%)	0 (0.0%)	0 (0.0%)	0 (0.0%)
Perceived health change (single item)	Control	0 (0.0%)	1 (3.7%)	0 (0.0%)	1 (3.7%)
Perceived health change (single item)	Experimental	0 (0.0%)	5 (18.5%)	0 (0.0%)	7 (25.9%)

Values are n (%); the denominator is 27 participants in every group. A score of 0 is the floor and 100 is the ceiling. Frequencies describe accumulation at the scale boundaries; they do not establish an intervention effect. Perceived health change is a single item.

**Table A3:** Diagnostic summaries and robust tests of differing baseline slopes.

Outcome	SD Residuals Control	SD Residuals Experimental	HC3 *p* Group × Baseline	Fitted Outside 0–100
Physical functioning	1.63	10.36	0.2757	6
Physical role limitations	12.10	25.99	<0.001	12
Emotional role limitations	7.90	18.11	0.0814	19
Vitality	6.53	11.48	0.0603	1
Emotional well-being	3.20	7.94	0.0772	1
Social functioning	5.81	12.65	0.6327	11
Bodily pain	7.78	15.62	0.1543	6
General health	3.21	7.13	0.1864	0
Perceived health change (single item)	8.82	21.93	0.0164	0

Residual SDs and counts of fitted scores outside the possible range are from the common-slope models. The group × baseline *p*-values are from interaction models with HC3 inference and 50 residual degrees of freedom; these are unadjusted diagnostic tests, not tests of the group effect at the baseline reference score.

**Table A4:** Residual distribution and influence in common-slope models.

Outcome	Shapiro–Wilk *p*	Maximum Cook’s D	*n* with Cook’s D > 4/54
Physical functioning	<0.001	0.150	5
Physical role limitations	<0.001	0.363	4
Emotional role limitations	<0.001	0.548	4
Vitality	0.0169	0.875	3
Emotional well-being	<0.001	0.573	2
Social functioning	<0.001	0.809	2
Bodily pain	<0.001	0.503	3
General health	<0.001	1.370	3
Perceived health change (single item)	<0.001	0.538	4

Shapiro–Wilk tests are descriptive diagnostics and were interpreted alongside residual plots. Cook’s D > 4/54 was used to flag observations for examination, not as an automatic exclusion rule. All participants remained in the full-sample analyses. Graphical diagnostics indicated departures from residual normality and greater residual variability in the experimental group.

**Table A5:** Leave-one-participant-out sensitivity of the different-slopes HC3 models.

Outcome	Full-Sample Effect	Range of Effects	Range of Unadjusted *p*	Iterations with CI Including 0
Physical functioning	3.92	3.02 to 5.62	0.0254 to 0.2455	53/54
Physical role limitations	23.28	19.72 to 25.03	<0.001 to 0.0072	0/54
Emotional role limitations	15.69	9.91 to 19.52	0.0192 to 0.5138	53/54
Vitality	10.39	7.64 to 11.33	0.0034 to 0.0141	0/54
Emotional well-being	7.58	5.80 to 8.05	<0.001 to 0.0030	0/54
Social functioning	5.22	1.73 to 9.74	0.0456 to 0.7285	53/54
Bodily pain	7.15	5.10 to 10.48	0.0061 to 0.2635	53/54
General health	3.98	3.11 to 4.56	0.0098 to 0.0379	0/54
Perceived health change (single item)	11.72	9.90 to 14.83	<0.001 to 0.0718	3/54

Each of 54 iterations omitted one participant and refitted follow-up score ~ group × centered baseline. The baseline reference remained the pooled mean from the original full sample. HC3 inference used 49 residual degrees of freedom in each reduced sample. The ranges are minimum and maximum across iterations; they are not confidence intervals. Iteration-specific confidence intervals and *p*-values were nominal and not multiplicity-adjusted. These checks assess single-participant influence and do not constitute independent confirmation of efficacy.

**Table A6:** Sensitivity of Holm adjustment to the HRQoL test family.

Analysis	Outcome	Unadjusted *p*	Holm 9 Outcomes	Holm 8 Domains
ANOVA	Physical functioning	0.2109	1.0000	0.8436
ANOVA	Physical role limitations	0.0240	0.1683	0.1443
ANOVA	Emotional role limitations	0.2115	1.0000	0.8436
ANOVA	Vitality	<0.001	0.0044	0.0039
ANOVA	Emotional well-being	0.0012	0.0094	0.0083
ANOVA	Social functioning	0.3152	1.0000	0.8436
ANOVA	Bodily pain	0.1361	0.8168	0.6807
ANOVA	General health	0.5634	1.0000	0.8436
ANOVA	Perceived health change (single item)	0.3549	1.0000	—
Common-slope	Physical functioning	0.1059	0.4236	0.4236
Common-slope	Physical role limitations	0.0060	0.0484	0.0423
Common-slope	Emotional role limitations	0.2149	0.4298	0.4298
Common-slope	Vitality	0.0094	0.0659	0.0564
Common-slope	Emotional well-being	0.0011	0.0101	0.0090
Common-slope	Social functioning	0.3335	0.4298	0.4298
Common-slope	Bodily pain	0.1156	0.4236	0.4236
Common-slope	General health	0.0190	0.1143	0.0952
Common-slope	Perceived health change (single item)	0.0369	0.1845	—
Different-slopes	Physical functioning	0.1475	0.4197	0.4197
Different-slopes	Physical role limitations	0.0015	0.0119	0.0104
Different-slopes	Emotional role limitations	0.1049	0.4197	0.4197
Different-slopes	Vitality	0.0072	0.0503	0.0431
Different-slopes	Emotional well-being	<0.001	0.0089	0.0079
Different-slopes	Social functioning	0.3850	0.4197	0.4197
Different-slopes	Bodily pain	0.1393	0.4197	0.4197
Different-slopes	General health	0.0190	0.1140	0.0950
Different-slopes	Perceived health change (single item)	0.0240	0.1202	—

ANOVA denotes the original mixed ANOVA group × time interactions; Common-slope and Different-slopes denote the baseline-adjusted ANCOVA models with HC3 inference. Holm adjustment was applied separately within each analysis. The nine-outcome family comprises eight RAND domains plus the perceived health-change item and is the main multiplicity approach for the revision. The eight-domain family excludes that item and is reported as sensitivity analysis; the dash indicates that no adjusted *p*-value was assigned to the excluded item in that family. Models are not independent confirmatory analyses. Conclusions should not be selected according to whichever family or model yields the smallest *p*-value.
